# Complete genome sequence of a human influenza a virus (H1N1) detected in Kazakhstan in the fall of 2022

**DOI:** 10.1128/mra.01040-24

**Published:** 2024-12-10

**Authors:** Radmir Sarsenov, Dmitriy Babenko, Dinara Kamalova, Asylulan Amirgazin, Rosaliya Begaidarova, Saule Atshabarova, Alexandr Shevtsov, Yekaterina Sidelyova, Sergey Yegorov, Irina Kadyrova

**Affiliations:** 1 Department of Biology, School of Sciences and Humanities, Nazarbayev University, Astana, Kazakhstan; 2 Research Centre, Karaganda Medical University, Karaganda, Kazakhstan; 3 National Center of Biotechnology, Astana, Kazakhstan; Queens College Department of Biology, Queens, New York, USA

**Keywords:** influenza, genomes, H1N1

## Abstract

An *Orthomyxoviridae Alphainfluenza virus Influenza A* virus strain, *A/Kazakhstan/Flu-H11-6/2022(H1N1*), was isolated in Karaganda, Central Kazakhstan during a study of acute respiratory infections among hospital inpatients in 2022. Here, we present the complete genome sequence of this strain.

## ANNOUNCEMENT

Influenza is an important cause of global morbidity. During most of the coronavirus disease 2019 pandemic (2020–2022), the Influenza A circulation dwindled in Kazakhstan (1–3), but it subsequently returned with higher transmission rates in fall of 2022 (4). H3N2 strains dominated globally during the 2022–2023 season, but A/H1N1(pdm09) strains predominated in fall of 2022 in Russia (4, 5).

In November 2022, we detected the strain *A/Kazakhstan/Flu-H11-6/2022*(*H1N1*) with nasopharyngeal swab collected from a hospitalized 3-year-old male patient with clinical symptoms of respiratory disease (6). RNA was extracted with MagMAX Viral/Pathogen Kit. Reverse transcription (RT)-PCR was performed using TaqPath 1-Step RT-qPCR, CG targeting Influenza A/H3N2, A/H1N1, and Influenza B strains (6) with a positivity threshold of <28. cDNA synthesis and PCR amplification were performed using the Superscript III One-Step RT-PCR System with Platinum Taq High Fidelity using multiplex RT-PCR primers for Influenza A virus (MBTuni-12 and MBTuni-13, Zhou [7]). Reverse transcription was carried out using the RNAscribe RT Kit, and the products were purified using Agencourt AMPure XP magnetic particle-based reagents. DNA libraries were prepared using Illumina® DNA Prep, Tagmentation (96 samples), and whole-genome sequencing was performed on the Illumina MiSeq platform using the MiSeq Reagent Kit v3 (600 cycles, acquired 352,430 paired-end reads, average length: 290 bp). Assembled contigs were identified via Local BLAST+ v2.12.0 (8) comparison with the NCBI Influenza Virus database and the highest nucleotide identity selected: MN061200, MN061201, MT211042, MT466208, MT211045, MT465985, MN061206, and MT211046, which were used as reference sequences for the alignment using BWA v0.7.17 (9), mutations identified with Free Bayes v1.3.7 (10), and consensus sequences generated with BCF (11). The completeness of segment coverage was verified in the IGV program (12) (default settings for all software were used, unless specified otherwise). This resulted in the assembly of a complete genome.

We performed quality control by utilizing FastQC v0.12.1 (13), trimming with SeqTK v1.4 (parameters: –b 20 –e 3) (14) and Sickle v1.33 (parameters: –t sanger –l 70 –q 30 -g) (15). Genome assembly was done with SPAdes v3.15.5 (parameters: --careful –k 127) (16). Finally, we used the NCBI Blast search of eight segments and selected 50 results with the highest identity to the *A/Kazakhstan/Flu-H11-6/2022(H1N1*) strain for the phylogenetic analysis. MAFFT v7 (17) was used to align the hemagglutinin and neuraminidase sequences, and phylogenetic trees were constructed using IQ-TREE multicore v2.0.7 (-bb 100000 -ntop 200 -nstop 5000) (18) with the K3Pu + F + I/GTR + F + I models chosen according to AIC/AICc, prioritizing the balance of complexity and data fit. The iTOL v5 tool (19) was used to visualize the resulting trees.

**Fig 1:**
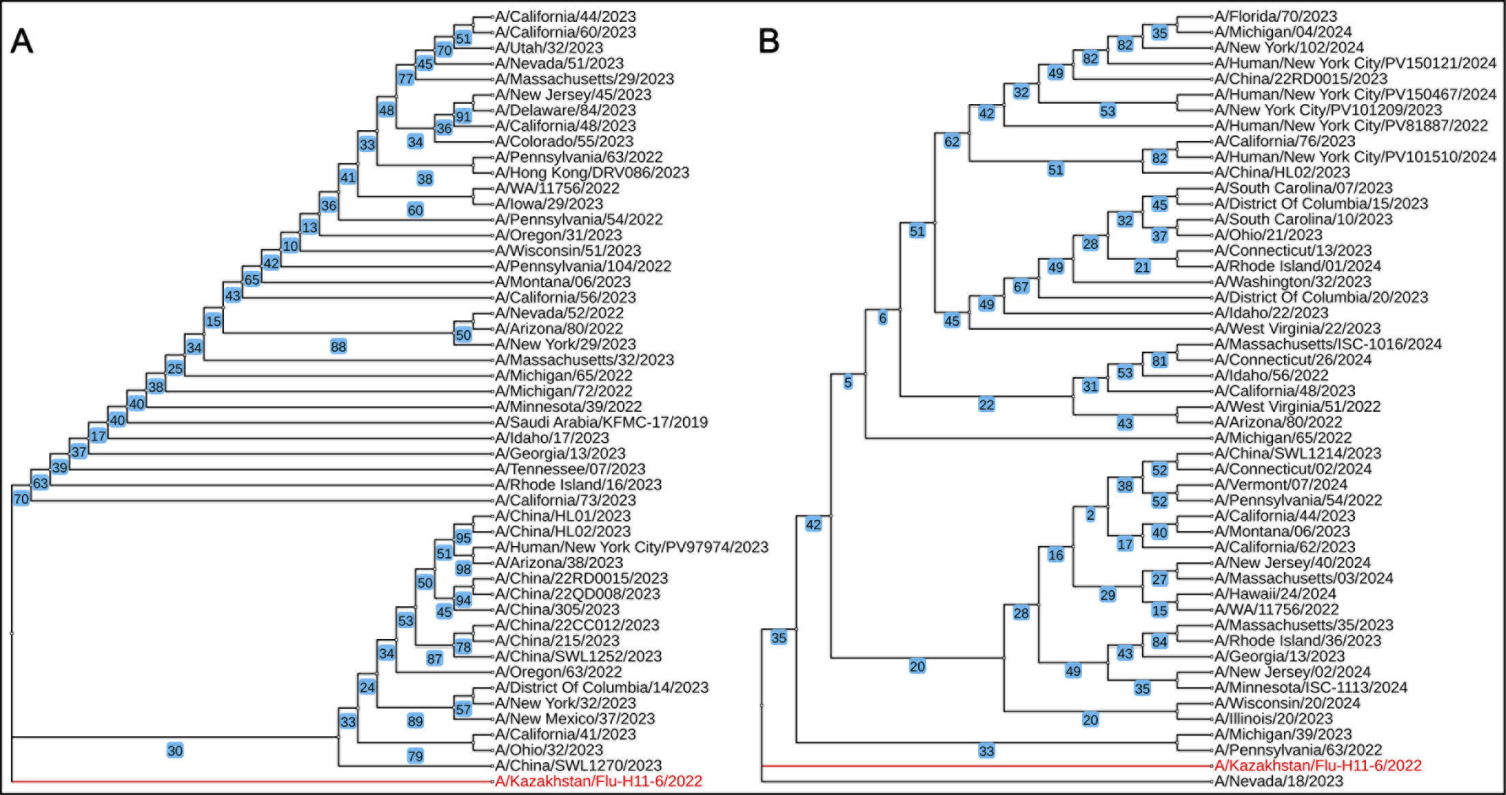
Maximum likelihood tree depicting the relationship between the hemagglutinin (**A**) and neuraminidase (**B**) genes of the selected Influenza A/H1N1 strains (*n* = 50). The isolated *A/Kazakhstan/Flu-H11-6/2022(H1N1*) strain is highlighted in red. The trees were constructed with 100,000 bootstrap replicates.

As shown in TableTable 1 and Fig. 1, the isolated *A/Kazakhstan/Flu-H11-6/2022(H1N1*) strain is most closely related (>99% nucleotide similarity, conclusion reached using BLAST) to A/H1N1 strains from North America and China.

**TABLE 1:** Comparison of the nucleotide sequences of all genes of the Kazakhstan Influenza A strain with the genetically most closely related strains in GenBank

Gene or segment	Size (nucleotides)	GC content (%)	Most closely related strain	Identity with most closely related strain at nucleotide level (%)	Query cover	GenBank accession #
Segment 1: Polymerase PB2 (PB2)	2333	44.11%	*A/China/22QD008/2023(H1N1*)	99.91%	100%	PP407268.1
Segment 2: Polymerase PB1 (PB1)	2332	41.04%	*A/Oregon/63/2022(H1N1*)	99.87%	99%	PP407269.1
Segment 3: Polymerase PA(PA)	2233	42.5%	*A/California/41/2023(H1N1*)	99.91%	98%	PP407270.1
Segment 4: Hemagglutinin (HA)	1752	40.81%	*A/Oregon/63/2022(H1N1*)	99.83%	100%	PP407271.1
Segment 5: Nucleocapsid protein (NP)	1565	45.24%	*A/Human/New York City/PV81887/2022(H1N1*)	99.94%	100%	PP407272.1
Segment 6: Neuraminidase (NA)	1433	41.45%	*A/Human/New York City/PV81887/2022(H1N1*)	99.93%	100%	PP407273.1
Segment 7: Matrix proteins 2 and 1 (M2, M1)	1020	45.98%	*A/China/22CC012/2023(H1N1*)	100%	100%	PP407274.1
Segment 8: Nuclear export protein (NEP)	890	42.47%	*A/China/22RG022/2023(H1N1*)	99.89%	100%	PP407275.1

All procedures involving human subjects were approved by the Commission on Bioethics of Karaganda Medical University under Protocol #12, Approval #45 dated 6 April 2020 and were conducted in compliance with the laws governing biomedical experiments and preclinical and clinical studies, as per Regulation No. 697 dated 12 November 2007 in the Republic of Kazakhstan.

## Data Availability

The complete genome of *A/Kazakhstan/Flu-H11-6/2022(H1N1*) is available at GenBank under the accession numbers PP407268.1 to PP407275.1. Raw sequence reads were deposited under BioProject accession number PRJNA1162313.
